# COVID-19 vaccination and menstrual disturbances: A prospective study from Pakistan

**DOI:** 10.12669/pjms.40.7.8709

**Published:** 2024-08

**Authors:** Nuzhat Parveen

**Keywords:** COVID-19, SARS-CoV-2, Vaccine, Menstrual cycle

## Abstract

**Objective:**

To evaluate whether or not immunization against COVID-19 is associated with changes in the duration and frequency of the menstrual cycle.

**Methods:**

This prospective analysis included the menstrual cycle data of 154 females after COVID-19 vaccination from August 2021 to March 2022. This study included Pakistani females aged 18 to 45 years and who had taken at-least one dose of COVID-19 vaccination. After two months of COVID vaccine the participants were interviewed again about the timing and duration of their menstrual cycle. The increase in menstrual length for >eight days was labelled as increased menstrual cycle duration.

**Results:**

Mean age of participants was 33.53±8.52 years. Among 154, 113 (73.4%) were married. Among 154 females, menstrual abnormality was reported by 59 (38.3%) females, increase in cycle duration was reported by 25 (16.2%) patients and decrease by 22 (14.3%), increase in number of bleeding days by 20 (13%) females and decrease by 15 (9.7%), increase in pain intensity was reported by 19 (12.3%) females and decrease by 17 (11.0%), increased intensity of blood flow was reported by 20 (13.0%) patients and decreased intensity by 19 (12.3%) females.

**Conclusion:**

COVID-19 vaccination is not associated with menstrual abnormalities in a significant number of females.

## INTRODUCTION

As a result of the COVID-19 pandemic, many investigations and publications have been carried out in different parts of the world in an effort to gain a better understanding of the scope of the disease as well as its short-term and long-term effects on the human body.^1^-^4^ The efforts have also been directed to find a cure for the illness.^5^

The development of vaccinations against the severe acute respiratory syndrome coronavirus-2 (SARS-CoV-2) and the coronavirus disease of 2019 (COVID-19) pandemic is a significant contributing factor in slowing its progression.^6^ During the course of last few year, a number of different SARS-CoV-2 vaccines were developed, and additional development of these vaccines is currently taking place on a global basis. Inactivated vaccines, adenovirus vector vaccines, and mRNA vaccines are the three basic types of SARS-CoV-2 vaccinations that are now available.^7^ According to the World Health Organization (WHO), more than one hundred different vaccinations have been developed, and 26 of these vaccinations have been tested and evaluated in phase III clinical trials.^8^ The vaccine monitoring agencies have brought attention to a variety of common adverse effects of the covid-19 vaccination. These adverse effects include a sore arm, fever, fatigue, chills, nausea, vomiting and myalgia.^9^

One of the many impacts that the COVID-19 vaccine has had is that it has had a detrimental impact on the health of women. Concerns about a possible connection between the immunization for COVID-19 and abnormal menstrual cycles can lead to reluctance to get vaccinated against the disease.^10^,^11^ This can be one reason why female patients are hesitant to get vaccinated.^10^ Unfortunately, the effects of vaccine on the menstrual cycle following vaccination were not recorded during the clinical trials of the COVID-19 vaccinations that are currently available, which was a major limitation of these researches.^12^-^14^ In this study, we offer an analysis of menstrual cycle monitoring data that were prospectively obtained from female population in Pakistan. The purpose of this study was to evaluate whether or not immunization against COVID-19 is associated with changes in the duration and frequency of the menstrual cycle.

## METHODS

This prospective analysis included the menstrual cycle data of 154 females after COVID-19 vaccination. The common vaccines used were Sinovac, Sinopharm, Pfizer, Moderna and AstraZeneca.

### Ethical Approval:

The study was conducted from August 2021 to March 2022 after taking approval from the Research Ethics Committee of the University of Ha’il [Nr.20455/5/42].

### Inclusion and Exclusion Criteria:

This study included Pakistani females aged 18 to 45 years and who had taken at-least one dose of COVID-19 vaccination and had passed at-least three menstrual cycles post-pregnancy or after use of hormonal contraceptives, and had normal menstrual cycle (24 to 38 days) and menstrual blood loss (considered normal by participants) were included for the study. The women using hormonal or intrauterine contraception devices, recent pregnancy (within three months), or breastfeeding were excluded from the study. Moreover, women with gynecological problems like severe menstrual pain, chronic pelvic pain, and those using any medication for cycle regulation in the past three months were excluded.

Data regarding women’s age and comorbid conditions was noted at the time of enrollment. The participants were enrolled from Rawalpindi region of Pakistan. After two months of COVID vaccine the participants were interviewed telephonically or via email by sending them the menstrual cycle related questions about the timing and duration of their menstrual cycle. The increase in menstrual length for >eight days was labelled as increased menstrual cycle. We also noted data regarding pain during menstruation, and intensity of blood flow.

### Statistical Analysis:

Data was entered in SPSS v25 software. Mean and SD was used to present continuous variables, number and percentage was used for presentation of qualitative variables.

## RESULTS

Mean age of participants was 33.53±8.52 years. Among 154, 113 (73.4%) were married. Only 8 (5.2%) patients had previous history of IUCD usage. Majority 60 (39%) patients were having no children. Majority of females 98 (63.6%) had master level education, and 45 (29.2%) had bachelor level education. Majority of females 93 (60.4%) were working. Only few females were having co-morbidities, among this anemia was the commonest one which was diagnosed in 29 (18.8%) females. Majority of females received Chinese companies-based vaccination, 70 (45.5%) had received Sinovac, 55 (35.7%) Sinopharm, only 15 (9.7%) received Pfizer, 10 (6.5%) AstraZeneca and 4 (2.6%) received Moderna (Table-I).

**Table-I:** Patient’s Baseline Characteristics.

Mean Age (Years)	33.53±8.52
***Marital Status***	
Married	113 (73.4%)
Unmarried	41 (26.6%)
History IUCD Use	8 (5.2%)
***Number of Children***	
0	60 (39%)
1-2	51 (33.1%)
≥3	43 (27.9%)
***Educational Status***	
Primary to Secondary Level	05 (3.2%)
High School Level	6 (3.9%)
Bachelor Level	45 (29.2%)
Master Level	98 (63.6%)
***Work Status***	
Working	61 (39.6%)
Non-working	93 (60.4%)
***Co-morbidities***	
Diabetes	07 (4.5%)
Hypertension	13 (8.4%)
Anemia	29 (18.8%)
Coagulation Disorder	13 (8.4%)
Thyroid Disorder	13 (8.4%)
Fibroids	10 (6.5%)
***Type of Vaccination Received***	
Sinovac	70 (45.5%)
Sinopharm	55 (35.7%)
Pfizer	15 (9.7%)
AstraZeneca	10 (6.5%)
Moderna	4 (2.6%)
***Number of Doses***	
1	42 (27.3%)
2	112 (72.7%)

Among 154 females, menstrual abnormality was reported by 59 (38.3%) females, increase in cycle duration was reported by 25 (16.2%) patients, increase in number of bleeding days by 20 (13%) females, increase in pain intensity by 19 (12.3%) females and increased intensity of blood flow was reported by 20 (13.0%) patients (Table-II). There was no significant association of type of vaccine with menstrual abnormality. Similarly, there was no significant association of number of doses of vaccine with menstrual abnormality (Table-III and IV).

**Table-II:** Menstruation after COVID-19 Vaccination.

***Menstruation Abnormality***	
Yes	59 (38.3%)
No	95 (61.7%)
***Cycle Duration***	
No Change	107 (69.5%)
Increased	25 (16.2%)
Decreased	22 (14.3%)
***Number of Bleeding Days***	
No Change	119 (77.3%)
Increased	20 (13.0%)
Decreased	15 (9.7%)
***Pain Intensity***	
No Change	118 (76.6%)
Increased	19 (12.3%)
Decreased	17 (11.0%)
***Intensity of Blood Flow***	
No Change	115 (74.7%)
Increased	20 (13.0%)
Decreased	19 (12.3%)

**Table-III:** Association of Type of Vaccination with Menstrual Abnormality.

	Menstrual Abnormality		P-value

	Yes	No	
Sinovac	30 (42.9%)	40 (57.1%)	0.15
Sinopharm	14 (25.5%)	41 (46.7%)	
Pfizer	8 (53.3%)	7 (46.7%)	
AstraZeneca	5 (50%)	5 (50%)	
Moderna	2 (50%)	2 (50%)	

**Table-IV:** Association of Number of Vaccine Doses with Menstrual Abnormality.

Number of Doses	Menstrual Abnormality		P-value

	Yes	No	
01	17 (40.5%)	25 (59.5%)	0.73
02	42 (37.5%)	70 (62.5%)	

## DISCUSSION

Alterations to the menstrual cycle can be caused by a wide variety of physiological and pathological factors, such as viral infections and changes in a person’s way of life. The menstrual cycle involves intricate interactions between a variety of tissues, hormones, and organ systems.^15^ Inflammatory components are involved in the majority of the physiological processes that occur in the female reproductive system.^16^ Cytokines and chemokines eventually become regulators of the environment of the uterus, where they play roles at various stages during the cycle.^17^ Endometrial tissue healing, angiogenesis, degradation, remodeling, and proliferation are some of the processes that are influenced by the inflammatory response.^16^

It is believed that COVID-19 is a condition that promotes inflammation, as it causes a cytokine storm and, as a result, causes the immune system to become exhausted.^18^ It has been reported that infection with SARS-CoV-2 can affect a woman’s menstrual cycle, regardless of whether or not she has been vaccinated against the virus.^19^ A study by Li et al. including 237 women, reported menstrual alterations in 177 (74.6%) women, among these 25% reported changes in bleeding volume, 28% irregularity in rhythm, 20% reported reduction in bleeding volume, 19% reported prolongation of cycle. The authors reported all these changes were transient and the cycle returned to normal after recovery.^20^

In order to become immunized against a disease, the body needs to produce an immune reaction. This immune response shows up clinically as mild post-vaccination side effects such as fever, headache, malaise, and gastrointestinal problems, but the end consequence is the formation of immunological memory. Induction of T cell activation that is capable of eliciting SARS-CoV-2 neutralizing antibodies is achieved with the COVID-19 immunization.^21^ Which intern can disturb the menstrual cycle temporarily.

In the present study, out of 154, 59 (38.3%) women reported changes in their menstrual cycle. Results documented that at least one of the four parameters indicated by FIGO to describe the menstrual cycle was outside the normal range after application of the vaccine and, therefore, determined an alteration in the menstrual cycle. Quejada et al. in a similar study on effects of COVID-19 vaccination on menstrual abnormalities including 408 women, reported menstrual disturbances in 184 (45.09%) patients. Among these 184, 65.21% patients reported prolonged duration, 41.84% reported increase in bleeding volume, 42.93% reported irregularity in interval between periods.^22^ Edelman et al. in their study on association of COVID-19 vaccination with menstruation changes reported that COVID vaccine is only associated with small changes in cycle length and it has no effect on menses length.^23^

In our study, we did not find any significant association of type of vaccination with menstrual abnormalities. Menstrual abnormality occurred in 37.5% females who received two doses of vaccine and in 40.5% females who received single dose of vaccine. Contrary findings were reported in the MECOVAC survey, the results of that survey concluded that the type of vaccination has no significant association with menstrual abnormality. The study reported that menstrual abnormality was more common among women who received two doses of vaccine as comparison to single dose group, the study also reported that the menstrual abnormalities returned to normal after two months of vaccination in half of the cases.^24^ Another study conducted in Pakistan reported that the incidence of manstrual disorders was higher among patients receiving Pfizer-Biontech, Sinopahrm COVID-19 vaccines with frequency rate of 14.9% and 9.5% respectively.^25^

This research precisely reported the menstrual cycle related changes that were brought up by the patients’ own self-reports. Concerns have been voiced about a variety of issues, including shifts in the length of the menstrual cycle and periods, variations in the symptoms associated with menstruation, unexpected bleeding, and shifts in both the quality and amount of menstrual flow. Self-reports are helpful for swiftly identifying potential signals or rare adverse occurrences; nevertheless, they have major limitations due to confounding factors and reporting biases, which prevents them from being fully reliable. One of the features of our study is that menstrual cycle data were obtained prospectively, which helps to decrease recollection bias.

### Limitations:

The major limitation of this study is that we collected patients only from single region of Pakistan, therefore sample may not accurately represent the entire female population in our country. We found significant number of females had menstrual disturbances after COVID-19 vaccine. The results thus demand regular collection of menstruation data in studies related to COVID-19 and vaccinations, as well as investigations into the mechanisms of menstrual disturbance following vaccination. To ascertain the percentage of women with long-term alterations, more study is required, including data on other menstrual health-related indicators and the assessment of bigger cohorts.
